# BIM-enabled built-asset information management conceptual framework: A case of public university buildings in Addis Ababa, Ethiopia

**DOI:** 10.1016/j.heliyon.2024.e33026

**Published:** 2024-06-13

**Authors:** Muluken Tilahun Desbalo, Asregedew Kassa Woldesenbet, Tamiru Mengist Habtu, Hans-Joachim Bargstädt, Mitiku Damtie Yehualaw

**Affiliations:** a Chair of Construction Engineering and Management, Bauhaus-Universität Weimar. Weimar, Germany; b Ethiopian Institute of Architecture Construction & City Development (EiABC), Addis Ababa University, Addis Ababa, Ethiopia; c Department of Construction Technology and Management University of Gondar, Gondar, Ethiopia; d Faculty of Civil & Water Resources Engineering, Bahir Dar Institute of Technology, Bahir Dar University, Bahir Dar, Ethiopia

**Keywords:** Building information modelling (BIM), Digital technology, Built-asset, Asset information management, Conceptual framework

## Abstract

The integration of BIM with other digital advancements has demonstrably led to an increase of performance in the Architecture, Engineering, Construction and Operation (AECO) industry. This integration not only is showing promising results in boosting the industry's performance, but also the productivity and promotes data-driven decision-making. Despite these benefits, there are limited studies that address the integration of BIM and digital data for managing built-assets in general and in developing countries in particular. To fill this gap, a closer assessment of current built-asset information management practice is necessary. The assessment of the practice examines how digital processes and/or technology can be seamlessly integrated into existing practices. In this regard, this study aims to provide valuable insights into increasing the maturity of built-asset information management by integrating digital data with BIM. The study uses a case-based research design using built-assets (universities in Addis Ababa) as public building representative to capture the prevailing information management practices in Ethiopian public buildings. The findings reveal that traditional paper-based practices still dominate the management of built-assets. This leads to difficulties in terms of data loss, unavailability, inaccuracy, and unreliability, all of which are detrimental to the overall performance. Based on these findings, a conceptual framework is designed to improve the performance of built-assets and help owners, end-users, and managers in defining data and information requirements for BIM-enabled asset information management. The framework delineates processes for structured information requirements definition and validation of various asset data from varying sources. The framework utilizes a BIM-enabled platform as a single source of truth and offers a comprehensive solution to the identified challenges. The findings of this study holds significant promise for improving the existing practice of built-asset information management within the study context.

## 1 Introduction

Due to the dynamic nature of work and business environment of the architecture, engineering, construction, and operation (AECO) industry, there is a growing need and expectation for better quality built-asset, improved performance and creating conducive environment. The emergence of the fourth industrial revolution has created a paradigm shift in managing built-assets, whereby traditional methods of operation & maintenance are being replaced by a more advanced approach [[1], [2], [3]]. One of the approaches is the delivery of relevant information [1,[4], [5], [6], [7]], as it plays a vital role in data-driven decisions to meet the rapidly changing needs and expectations of end-users of facilities [8].

Nonetheless, the lack of reliable and accurate data and/or information as a single source of truth is still a challenge in effective management of built-assets [[9], [10], [11]]. To alleviate this challenge, significant technological advancements are being utilized to support decision-making using digital information of built assets for improved operational performance. The emergence and advancement of digital technologies like Building Information Modelling (BIM), Artificial Intelligence (AI), Internet of Things (IoT), Laser Scanning, Digital Twin (DT), Virtual Reality (VR), Augment Reality (AR) and Block Chain (BLC) are good examples. These advancements are playing a significant role in facilitating data-driven decision-making processes and automate operational activities of built-assets within the AECO domain [[12], [13], [14], [15], [16]].

However, there is still practical challenges in terms of application and finding studies on the problem and lack of accurate data and information for managing existing buildings [[10], [11], [9]]. In developing nations like Ethiopia, decision-making without relevant & accurate data and information is common and consequently leading to poor management and operation of built-assets [[17], [18], [19]]. For instance, the lack of as-built documents of an existing building may lead a maintenance worker to damage a structural element that may jeopardize the building performance. The use of ground penetrating radar, concrete scanners and sensors for automatic fault detection are technological solutions that are developed to address such challenges. In addition, there are concerns and gaps on procedures/criteria to follow; method by which data should be generated; and the configuration as well as the level of specificity to capture information from built-assets efficiently [20].

Furthermore, the practical constraints required to be addressed are of two-fold. The first is related to meeting societal demand of built-assets due to the rapid urbanisation and population growth, according to United Nations [21]. The second is associated with operating and maintaining built-assets to provide the intended service to the core function. Thus, this study explores on the generation and delivery of accurate and reliable data for improving information flow in making data-driven decisions in built-asset management (BAM). In this regard, the study contributes to the development of strategic built asset management practices through utilisation of a bottom-up approach as well as encouraging the dissemination of knowledge and good practices from research institutes to the industry.

In light of this, the study aims to determine the type, level of detail and categorisation of information required to effectively integrate BIM into the operations and maintenance (O&M) decision-making process. The study uses a mix of qualitative and quantitative research methods and focuses on data collected from specific cases; especially public buildings represented by universities in Addis Ababa. Through this investigation, the study seeks to shed light on BIM-enabled asset information management by taking public buildings as case studies in Ethiopia and thereby make empirical contributions to the existing body of knowledge in the field.

The reminder of this paper consists of five parts: Section 2 discusses the state of the art and research gap in the domain; Section 3 describes the research design and methodology for thematic analysis and synthesis; while Section 4 presents the results of the study; and Section 5 presents discussions based on results; followed by a conclusion in section 6.

## 2 Literature review

### 2.1 Buildings in public universities and information management

Buildings in public universities are designed to serve the public, in terms of educational, research, and administrative services in a sustainable manner. Velazquez et al. [22],p.872 defines sustainable university as “*a higher educational institution, as a whole or as a part that addresses, involves and promotes on a regional or a global level. it minimizes negative environmental, economic, societal, and health effects generated in the use of resources to fulfil its functions of teaching, research, outreach and partnership, and stewardship in ways to help society make the transition to sustainable lifestyles*.” Because a built-asset in a university is complicated, multi-functional, and serves a substantial number of users, it involves a variety of operation & maintenance decisions. As a BAM or FM is a new profession, there is a limited pool of literature on the practice of public building asset management, particularly in developing countries like Ethiopia. On top of that, the local practice suffers from the variability of the universities in terms of organizational structure, operation methods, and functions of the BAM unit, which in turn is determined by the age of buildings, the size of the university; number of campuses; student enrolment and use of innovative technologies [23].

Given the research gap of utilising processes & technologies like Building Information Modelling (BIM) for existing buildings and the practice in the study context, there is great demand for studies in the overlooked sector of BAM, where BIM has high potential in digitizing an asset's lifecycle process to address the challenges. Both Ashworth et al. [20] and Shaw et al. [7] emphasized the critical need for operational information required by BAM teams to optimize operational & maintenance functions. This aspect has not been adequately addressed through the integration of BIM-BAM knowledge domain. Specifically, it was found that lack of attention to the information required by operation teams to effectively manage a built asset for optimal performance. This oversight underlines the importance of addressing operational information requirements within the BIM-BAM framework to enhance BAM practices. Numerous authors have also argued that it is imperative to identify pertinent information needed by the operational team early in the planning and design phases to ensure effective utilisation and decisions throughout an asset's useful life [1,7,24].

The importance of the successful transfer of data and/or information to operational information management platform such as a computer maintenance management system (CMMS), Computer-aided Facility Management (CAFM), and Building Automation system (BAS) are good examples [7,24,25]. The studies illustrated the importance of involving owners and operation team early in the process to ensure maximum value for organizational strategic goals. The operation and maintenance phase requires structured and organized data and information to support a data-driven decision-making process in asset management.

Operation and maintenance management phase is one of the most important, capital intensive and usually the longest life span compared with other phases within AECO [[26], [27], [28], [29], [30], [31], [32], [33]]. However, low attention is given to the post-construction stage or operation & maintenance management in the built environment in general [7,24]. This phase is important as it requires structured & organized data and information is needed to make data-driven decisions. The availability of high-quality data/information is vital to foster the management of operation & maintenance and maximise the value of built-assets [1,[4], [5], [6], [7]].

### 2.2 Information management for the performance of built assets

The advancements in Industry 4.0 technologies have catalysed a revolution in traditional information management practices in BAM and FM. This technological progress has accompanied in a significant transformation, greatly enhancing BAM/FM performance across various domains [2,3]. Booty [34] underscores that 90 % of operation & maintenance management functions are intricately tied to information management, whereby only 10 % involve engineering tasks. This emphasizes the pivotal role of information management in BAM performance, highlighting its undeniable importance in optimizing operations & maintenance activities. Nevertheless, the lack of accurate and reliable information poses a practical challenge to the integration of BIM into the BAM [[10], [11], [9]]. The information flows across the built asset lifecycle stages and within the operation team, is also poorly defined and structured.

Despite the challenges, BIM-BAM integration is growing rapidly because of its potential to improve performance and facilitate easy access to data in decision-making [35]. Even in developing countries, the adoption of BIM is increasing as it brings benefits throughout the life cycle of asset delivery [17,[36], [37], [38]]. Thus, there is a continuing need to analyse and align its influence with the profession's current level of development, keeping in mind the essential concepts of integrating people, place, process, and technology. In addition, digital technologies play a crucial role in BAM practice in sourcing, recording, integrating, modelling, storing, translating, and transforming information. Effective communication and a smooth flow of information are critical to achieving BAM's strategic goals, which is widely recognized as an interdisciplinary approach to facility management [23,39,40].

### 2.3 Integration of Building Information Modelling (BIM) with built-asset management (BAM)

One of the requirements for a coordinated built asset management (BAM) practice is information logistics for effective and data-driven decision-making. BIM can potentially improve BAM practice by extending the model into the post-occupancy period and providing a robust information platform [8]. BIM-BAM data integration comes with establishing an effective process to extract, store, and distribute data to ensure interoperability [41]. Although the management of graphical, non-graphical, and document-based information of an asset from feasibility to construction phase through BIM-BAM based practices is widely adopted, the integration of such practices has not been sufficiently integrated with BAM systems [7,20,42]. BAM requires interoperable information management platforms for existing buildings to support capturing, storing, and integrating data with BIM. A platform capable of storing, transferring, and translating BAM data for key decisions is in high demand, with BIM emerging as ideal solution [41]. However, challenges remain, including insufficient integration from the project strategy to the closeout phase. This can be attributed to lack of standardization of information exchange formats, variable data formats & sources; big data (size) and poor performance in automating information exchange between authoring tools. These challenges significantly hinder the process and practice of asset information management.

### 2.4 BIM for existing buildings

The implementation of BIM for existing buildings is relatively a new trend. There is still uncertainty about the real benefits of information modelling of already built-assets [43,44]. The idea of BIM still sounds too many implying BIM as an unnecessary technical innovation, which can have limited impact on a business while there are other reliable information management systems in place. In addition, implementing BIM to create a model of an existing building is challenging than designing a new building from scratch, whereby the concept of reverse engineering can be valuable [23]. Over the last decade, however, research into the integration of BIM with built asset management has gained traction, with the associated business benefits becoming the focus of research and gaining widespread acceptance. The emergence of BIM increases the efficiency of BAM by enabling comprehensive information capture throughout the lifecycle an asset [5,6,45]. The lack of BIM-based modelling and accurate as-built drawings in many existing buildings underlines the critical need for BIM integration. While this endeavour is essential, it often proves to be complicated and financially costly [6,46]. As asset management (AM) aims to achieve the owner's objectives by balancing costs, opportunities and risks, reliable asset information is essential for effective decision-making, planning and execution. This need is particularly pronounced in operations and maintenance, as emphasized by Carbonari et al. [47], ISO 55000 [48].

BIM evolution not only improves design and construction processes, but also asset management by streamlining preventive and scheduled maintenance through real-time data access, verification, and management [10]. The integration of BIM into built assets benefits asset owners by reducing expenses, optimizing maintenance, and providing organizational and strategic benefits [6,49,50].

**Fig. 1:**
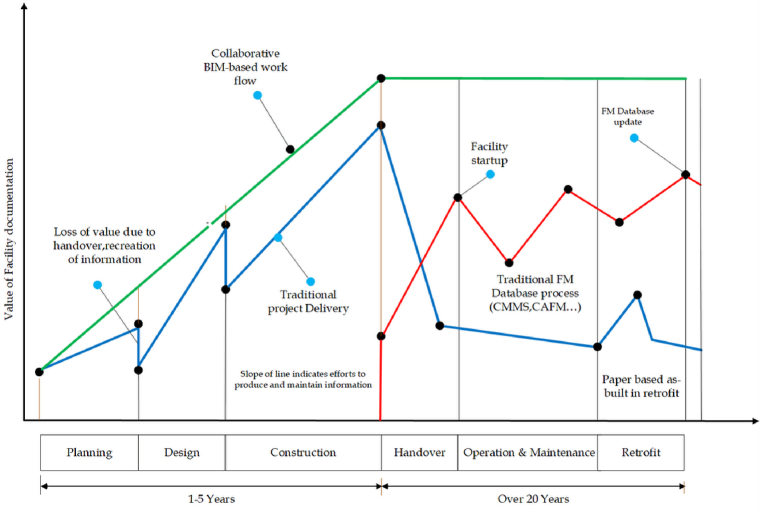
Representation of Value of Information Across Asset's Lifecycle (Elaborated based on Eastman et al. [33]).

Numerous scholars emphasize the benefits of integrating BIM with built assets, including improved collaboration, data management and decision making, leading to more efficient maintenance operations [6,33,[50], [51], [52]]. BIM-based built asset management improves the accuracy, completeness of building data and visualization capabilities, overcoming information loss and data asymmetries and ultimately improving asset management processes [4,6,51]. Fig. 1, ‘Islands of automation in construction’ Hannus et al. (1998, cited in Eastman [33] visualizes the opportunities and barriers of sharing building data and illustrates the value of information across the lifecycle of assets and how BIM minimizes information loss and improves the value of assets over time. BIM offers potential cost savings by enhancing operational phase functions [4,6,[50], [51], [52], [53], [54], [55]]. Integration of BIM with BAM facilitates efficient building commissioning and accurate information handover for management databases [1,4,16,33,45,51]. However, the challenge of information loss between phases incurs additional costs, highlighting the importance of information integrity throughout the asset Lifecyle [18,33,56]. This underscores the increasing value of information downstream in the operational phase.

### 2.5 Information requirements

Information requirements are the sets of information needed to support the process activities in the particular phase of a product lifecycle [57]. At the critical phases of a project, information requirements are released by an owner's assessment and approval [58]. In the international BIM standard BS EN ISO 19650–3 and BS EN ISO 1960–1, information requirements are inputs for the whole information management, which includes organizational information requirements (OIR), asset information requirements (AIR), project information requirements (PIR), and employer/exchange information requirements (EIR).

**Fig. 2:**
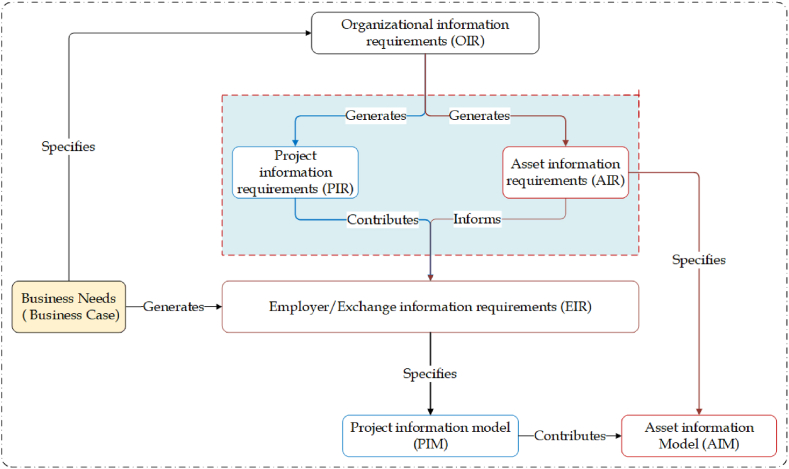
Elements of BIM-based Information Management. (Elaborated based on [9,63].

Therefore, the responsibility of an asset owner lies in articulating and converting strategic information requirements (OIR) into operational information requirements (AIR and PIR), and subsequently integrating them into the tender specification (EIR). This process is detailed by scholars such as Barnes [59], Building and Construction Authority [60], and Pavan et al. [61]. OIRs are the information required by an organization for asset management and operation for informed decision-making about high-level strategic objectives [26,60,62]. On the other hand, asset information requirement (AIR) is a part of a BIM process that incorporates the required graphical and non-graphical data/information and documents of the built-asset at the O&M phase (ISO-19650-1, 2018). According to BS EN ISO 19650–1 Guidance Part 4 standard, AIR is an information requirement specification for “what”, “when”, “how”, and for “whom” information is to be produced. The integration of information requirements that are in line with an organisation's core business objectives is illustrated in Fig. 2.

### 2.6 Operational phase information requirements

There exists a mismatch between the type and amount of relevant information that is needed by BAM staff and what is currently being delivered by design and construction professionals in built-asset handover [4,20,57]. In asset lifecycle, there is a generalized failure while transferring information between the development (design and construction) and operational phases. The steady uptake of digital technologies, such as BIM in the design and construction phase has been accompanied by an expectation that it would enable a better transfer of information to those responsible for built asset management [4,44,64]. The management of built-asset information often involves unreliable, slow, and costly procedures to a great extent, based on printed handover documents [4,27]. This information can be integrated with BIM to improve information utilisation and thus reduce mistakes and reworks in renovation/revitalization projects [14,65]. The flexibility of new designs in specifying components and their locations is an advantage which cannot be attained in information modelling for existing buildings as it requires accurate representation of existing conditions.

Because the components have been in service for a long time, they may demonstrate physical durability, deformation, and corrosion [23,46]. Scan-to-BIM can be used in this context, as it offers a robust technological solution for capturing 3D actual data of existing buildings.

### 2.7 Scan-to-BIM for existing buildings in asset information modelling

Generating as-is BIM models retrospectively is an essential process in asset information model for existing assets. While the presence of these models is essential in the implementation of BIM, it is often the case that either a suitable model is non-existent or is less relevant or outdated for existing buildings [66]. Consequently, there is a strong need to retrospectively create digital models for existing buildings. Scan-to-BIM is a process of capturing accurate data from existing objects or buildings into BIM models [67]. Scan-to-BIM involves various scanning technologies ranging from 3D laser scanning, unmanned aerial vehicles, such as Light Detection and Ranging (LiDAR), and mobile devices to generate 3D point cloud data [66,[68], [69], [70]].

The selection of these equipment and devices depends on several factors such as cost, scope of work, accuracy, operation time, processing time workflow, and quality [71], availability of equipment and expert. Once a 3D point cloud data is captured, the data can then be transformed into BIM model for further processing. Scan-to-BIM can also be used for assessing the accuracy and precision of generated BIMs, helping decision-makers evaluate the risk of decisions based on the information [66,70]. In addition, scan-to-BIM can be used to evaluate retrofit plans for existing buildings based on energy efficiency and renewable energy targets [66,67,70].

### 2.8 Asset information management practice (The study context)

Asset owners, in the context of the present study, receive manual asset data, if it exists from different sources at different phases of project lifecycle as stated in Ref. [72]. It is of utmost significance to ascertain that the information and data that have been handed over, emanating from diverse sources, are in congruence with the framework of an owner's asset management system [26,73]. In this regard, the establishment of efficient BAM practices within public buildings is of paramount importance.

Given the recent mandate requiring the registration of new built-assets as a means of evaluating post-occupancy performance, the identification and classification of public assets is now deemed a matter of urgency in the context of the present study [74]. As a result, owner shall include in the structure of asset registry, list of asset type, attributes and database taxonomy and other relevant constructs for effective implementation of the proclamation and enhance performance of the assets and employee's productivity. The effective introduction and implementation of new technologies to maximise the use of information is a major obstacle, especially for large public asset owners. In establishing a BAM system, asset definition needs to be considered by owner internal departments including built asset type, source of asset data, asset value and associated cost of operation and maintenance work orders for each asset need to be assigned [72]. To overcome the challenges associated with built asset information management in the context of the study, it is essential to apply a rigorous scientific methodology to ensure the validity of the results obtained.

## 3 Research design

### 3.1 The study design

This study adopted a mixed method of quantitative and qualitative data using distinct designs for philosophical assumptions and theoretical frameworks. This approach yields dependable and authentic findings [75,76]. The qualitative study is categorized as exploratory and descriptive due to the absence of prior research in the study context. Additionally, considering the nature of the problem and limited data availability, a comprehensive contextual investigation is necessary. Thus, a case study is selected as research design focusing on public buildings in three universities in Addis Ababa, Ethiopia, and utilises semi-structured and structured interviews for qualitative analysis as an integral part of the study. A carefully crafted set of interrogative guidelines were designed to investigate the significance of each information classification and sub-classification provided by the interviewees in the case studies.

Case studies, in their true essence, explore and investigate contemporary phenomena through detailed contextual analysis of a limited number of events/conditions and its relationships within real life [77]. The selection of the cases is based on inclusion of a mix of representative old and new universities within the city (assuming a relatively better experience in asset management in the old ones), to conduct further investigation and observations, availability of various building types, including university teaching hospitals and other off campus building assets. The quantitative approach involves an examination of the frequency of information requirements revealed by interviewees during structured interviews. Subsequently, the significance of these requirements is assessed through the utilisation of equations (1), (2)), with weighting applied to accommodate responses from all participants across the three case studies.

(1) $${\bar{x}}_{ik}=\frac{1}{N}\;\sum_{i=1}^{n}x_{ij}$$

$x_{ij}$ denotes the response of jth interviewee to particular question in case studies and i = 1,2,3 ….n where n is the number of questions. ${\bar{x}}_{i}$ is the mean response for questions i in case study k, which ranges for 1–3 and N represents the number of interviewees who participated in each case study.

The overall average response, w, to question I across all three case studies can be calculated as the average of ${\bar{x}}_{i},{\bar{y}}_{i}and\;{\bar{z}}_{i}$ equation (2).

(2) $${\bar{\omega }}_{i}=\frac{1}{3}\;\left({\bar{x}}_{i}+{\bar{y}}_{i}+{\bar{z}}_{i}\right)$$

Where ${\bar{\omega }}_{i}$, is the overall average response for question i, across the three case studies, ${\bar{x}}_{i}$ is the mean response for question i in case study 1. Similarly, ${\bar{y}}_{i}and\;{\bar{z}}_{i}$ represent the mean of responses for question i in case studies 2 and 3, respectively.

### 3.2 Sample size and selection criteria of cases

Sample sizes in case studies are typically small, which is common in most qualitative research [28]. Yin [78] argued the existence of situations when investigating a case can be extremely important. Theorizing on the basis of multiple cases can be done effectively with a small number of cases, usually between four and ten [79]. This method involves collecting comprehensive data from multiple sources to formulate robust, emergent theories that are precise, coherent, and concise. The iterative process of case selection, data analysis and hypothesis development ease the creation of innovative & empirically grounded theories, although challenges such as complexity and narrow focus may arise in the resulting theories. Each individual case is a unique experiment that contributes to the development of a more comprehensive theory.

If a single case behaves similarly to cases in the same situation, it provides evidence of its strength and the credibility of its findings. Using such a case is analogous to performing a test after many tests. It serves as a benchmark or reference point that allows researchers to increase their sample size for replication purposes without compromising data quality. A larger sample increases the research's validity by minimizing the likelihood of security incidents or other irregularities affecting the results. While using multiple cases or sampling within a case, purposeful sampling is effective. This study employs a multiple-case study design, following the recommendation by Yin [78].

Accordingly, a pragmatic approach is taken considering documents from practice in line with guidance from Saunders et al. [75] and supported by detailed exploration of the case study as utilised in Ashworth et al. [20]. The documents reviewed included standards, articles, strategic plans of public universities, manuals, reports, national proclamations, web pages, and organizational documents.

### 3.3 Justification of selected cases

According to the educational statistics of the Federal Ministry of Education of Ethiopia, MoE [80], the country had only six (6) public universities and eleven (11) colleges and institutes in 1999/2000. However, forty-four (44) new universities and upgrading of existing colleges to university level have brought the total number of public universities to fifty (50) by 2020 [23,81].

As a result, the government made significant investments in creating new universities and expanding existing ones to deliver higher education as an integral part of a University Capacity Building Program (UCBP) [35].

The construction of these university facilities included student dormitories, libraries, lecture halls, classrooms, offices, student cafeteria, laboratories, assembly halls, and staff residence. In the years 2018, 2019, 2020, and 2021, the government's budget spending for the projects has grown significantly, reaching 25, 22, 26, and 28 billion Ethiopian Birr, respectively (equivalent to more than 520 million USD per year) [82].

Despite this huge investment, the projects have encountered several challenges that have extended beyond the development phase and have landed in asset management phase. As-built data & documentation is one of the critical challenges (should have been handed over by developers to the owner upon completion), which is a mandatory requirement included in the national general condition of contract [83]. Nevertheless, as-built documents are either incomplete, kept in hard copy (traditional paper-based), or lost during project handover as illustrated in Refs. [35,84].

In addition, there is a lack of knowledge and awareness of well-established built asset management practices and processes (working procedures, proper preventive maintenance schedules, and maintenance manuals) and other pertinent asset information to support a data-driven decision-making process. As a result, it has created difficulties the operational phase functions [85].

### 3.4 Study validity and reliability measures

Reliability and validity are concepts used to evaluate the quality of research. While reliability focuses on the consistency of a measure, validity assesses the accuracy of a measure [23]. A measurement can be considered reliable without being valid. However, if a measurement is valid, it is usually also reliable [86]. This study uses validity as a measure of quality. According to Yin [78], construct validity, internal validity, external validity, and reliability are the four-research quality measurements that can be used to judge the quality of any given case design Table 1.

**Table 1:** Validity and reliability in case study research, adopted from [23,78].

Tests	Case Study Reliability & Validity Checks
Construct validity	• Use of multiple sources of Evidence (Primary and secondary sources including reviews and multiple sources of data) • Have key informants review draft case study report
Internal validity	• Do pattern matching (Used in the analysis of transcribed interview) • Do explanation building (detail explanation is given for every finding)
External validity	• Use replication logic in multiple case studies (the presented results can apply to challenges in similar context)
Reliability	• Use case study protocol and procedure. • Test reliability of data collected using guideline (Cronbach's α was found to be,0.986) for the quantitatively analysed level of importance of information

In the present study, construct validity using document analysis and external validity using literal replication logic are adopted to check the research quality.

**Fig. 3:**
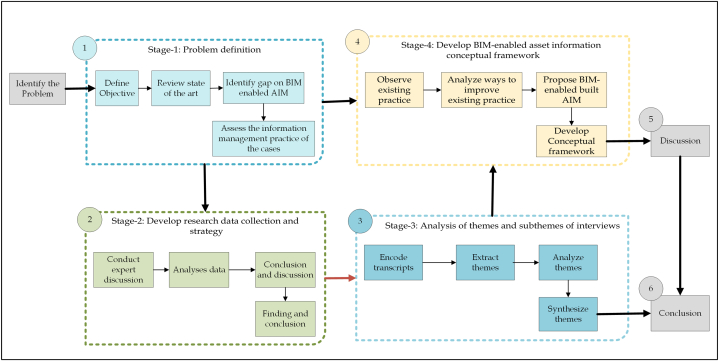
Flow of research process.

The overall research design followed in the development of the study is illustrated in Fig. 3. The overall research flow involves six main parts: Part one delineates the process of problem identification and the method employed to scrutinize the current information management practices of public universities in Ethiopia with a particular focus on selected cases. Part two illustrates the systematic procedure employed to gather data using quantitative and qualitative approaches via structured and semi-structured guidelines, encompassing desk study and interview. Part three describes the meticulous process of transcribing and encoding interviews and sorting quantitative data for further analysis and synthesis. Part four presents the steps followed to develop an asset information management conceptual framework corresponding to the present challenges. Part five delineates the discussion process conducted regarding the developed conceptual framework. Part six culminates the processes by providing a comprehensive conclusion.

## 4 Results

### 4.1 Sample characteristics

Table 2 illustrates a comprehensive overview of the case studies (public university buildings) included in the current empirical study.

**Table 2:** Case studies.

Case	Location	Year of establishment	No. of Campuses	No. of academic staff	No. of None-academic staff	No. of UG students	No. of PG students
1	Addis Ababa	2003	1	472	391	>8000	7000
2	Addis Ababa	1942	14	3110	4346	>29,872	>17,738
3	Addis Ababa	1988	3	>280	>1000	>8000	>7000

The analysis and discussion section are underpinned by a series of expert interviews, which serve to complement the three case studies. Table 3 shows a comprehensive overview of the demographic information of the interviewees in the three case studies.

**Table 3:** Demographic information of interviewee.

Cases	Interviewees/Participants	Roles	Year of Experience	Duration of Interview
1	P1	Institutional development and business vice president	>20	20 min
	P2	Directorate of project office	13	50 min
	P3	Directorate of facility management	12	61 min
	P4	Team leader for maintenance	6	61 min
2	P1	Directorate of facility management	12	61 min
	P2	Senior civil engineer	12	102 min
3	P1	Civil engineer	8	102 min
	P2	Directorate of project office	16	51 min
	P3	Directorate of facility management	12	51 min

*P1–P4 represent participated interviewees.

The interviewees have a wide-ranging knowledge and specific experience in the AECO domain. Work experience of 11–15 years is witnessed for about 60 % of the interviewees, while 22 % have more than 16 years. One way of measuring respondents' level of understanding of the study question is their work experience in public building development and management. Consequently, interviewees have adequate knowledge and exposure, as shown in Table 3. Furthermore, experts from the project office have also been identified as having a sufficient understanding of the problem due to their involvement in the upstream initial phases of the asset's lifecycle.

### 4.2 Analysis of current practice of built asset information management

The current practice of asset information management is examined to propose a solution in addressing existing complications. As a result, a qualitative thematic analysis of transcribed interviews is performed and the findings are presented in Table 4. Based on the results of both quantitative and qualitative methodologies, it has been explained that the instances included within the present study are considerably deficient in employing the latest technological advancements currently utilised in developed nations that contributed to getting optimized values from existing buildings. The lack of a systematized framework for information management, a database for asset registry, and the cumbersome finding of asset data have significantly hindered the implementation of data-driven decision-making and affected built asset management performance.

**Table 4:** Summary of Qualitative Data Results using Thematic Analysis (Interview).

Analysis Questions	Frequency of Appearances
Built-Asset registry practice (number of buildings, type, and function)	
• Asset registry practice is non-existent	**7**^a^
• Asset registry practice somehow exist and immature	1
**Availability of asset information and documents for existing buildings**	
• Asset information and documents are not available at all	**5**^a^
• Traditional paper passed documents partially available	**3**^a^
**Presence of as-built documentation for existing buildings**	
• No as-built drawing for old and recently completed Buildings	7^a^
• Paper based as-built drawings for recently completed buildings	1
**Asset data and maintenance operation document management system**	
• Paper based traditional document management	**5**^a^
• Database is needed but not available	3
• Lack of databases is stated as a major challenge for O&M functions	**5**^a^
**Data structure and Building classification system**	
• There exist no data structure within the operation team department	**8**^a^
• No building classification practice in general	**6**^a^
• Traditional classification of buildings	2
**Maintenance work orders management practice**	
• Totally traditional paper based, and loss of information is a challenge	**8**^a^
**Data capturing approach and defect identification method**	
• Traditional visual inspection	**8**^a^
• Non-use of technological equipment for data capturing and defect identification	**8**^a^
**Data file exchange format from project to operation team**	
• Fully Paper based and scanned digital files (drawings & documents)	**3**^a^
• Digital files with either/or (.DWG, DOX, PDF) formats	**3**^a^
• Combination of paper based and digital files	**5**^a^
• Preference for interoperable data exchange format for future use	**8**^a^
**Impact of data unavailability on the performance of O & M activities and process monitoring**	
• Main cause for rework in maintenance activities	**8**^a^
• Causes waste of time due to insufficient knowledge about a built asset	**8**^a^
• Difficulty of performance evaluation and monitoring of the O&M team	**8**^a^

a All Bold values are showing at least three interviewees stated the sub themes.

As illustrated in Table 4, the current practice of BAM in the context under study is characterized by a remarkable lack of modern tools and procedures. There is no asset register and there is a lack of important documentation such as as-built drawings for old and recently completed buildings. Relying on traditional, paper-based methods, the risk of information loss is high. This is compounded by the use of traditional visual inspection techniques and the lack of technological tools for data collection and defect detection. This outdated approach not only leads to frequent rework in maintenance activities, but also to lost time due to an inadequate understanding of asset details. In addition, the lack of effective performance evaluation tools makes it difficult to monitor and optimize the efficiency of the operations and maintenance team.

**Fig. 4:**
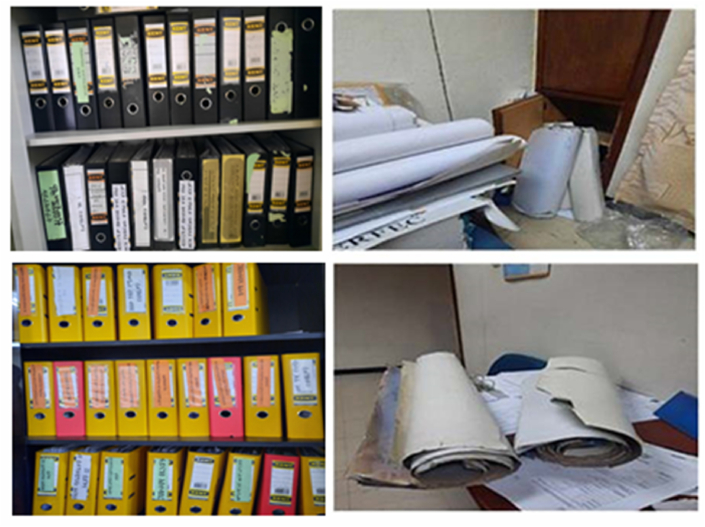
Traditional Paper-based Built-Asset File Storage. (Source: Photo taken during observation of the case study sites).

Overall, these factors underline the urgent need to modernize and digitise asset information management practices to enhance efficiency and effectiveness. Furthermore, Fig. 4 also illustrates a typical and traditional paper-based practise of managing built-asset data in the context of the study, which has exacerbated the need to digitise the process.

**Fig. 5:**
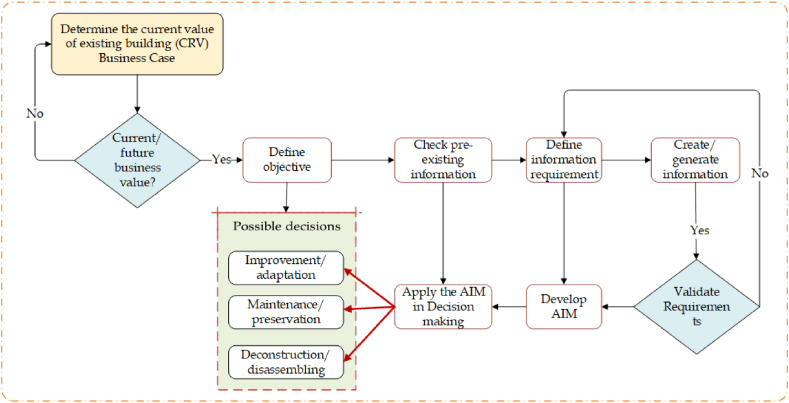
BIM model creation processes for existing building.

Despite the BIM potential for initial phases, its benefits in BAM have not been fully realised yet. Most existing buildings do not have a BIM model and creating a BIM for existing ones can be challenging [5,46,87]. While BIM processes are established for new buildings, the majority of existing buildings are not maintained, refurbished, or deconstructed with BIM yet [14,43,65,88]. Depending on the availability of previously existing BIM model, an update or new model can be considered for existing buildings. The process of creating a model from an existing building is explained based on key operational phase decisions, as depicted in Fig. 5.

The process in Fig. 5 aims to develop an efficient asset information model for existing buildings, considering the current business value of the asset. The data generation process is proposed to be based on current replacement value and potential (future) for a business case, which is consistent with potential decisions about a particular built asset, such as improvement/adaptation, maintenance/keeping or deconstruction/disassembling. This model is expected to maximise the value of the asset and be consistent with an organization's objectives. The improvement/adaptation, maintenance/preservation and deconstruction/disassemble decisions are identified through key operational decisions on building asset [35,[89], [90], [91], [92]].

### 4.3 Summary of information requirements

The information categories and examples of data types of the operational & maintenance team requires as determined from literature and ISO 19650–3 [63] for BIM-enabled asset management are presented in this section. In a BIM-based project, the required information can be generated from a project information model and organizational information requirements are aligned with corporate goals. The major types identified from the desk study are basic, technical, managerial, commercial, financial, and legal information requirements.

**Fig. 6:**
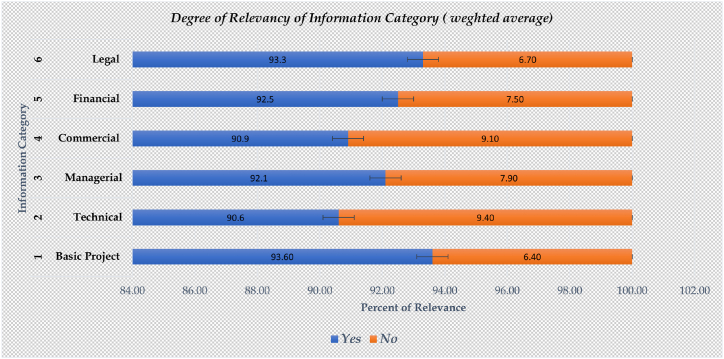
Summary of degree of information relevance.

Based on this category, interviewees rated the level of information relevance using a 10-point scale, one (1) being extremely irrelevant and ten (10) being extremely relevant. A structured questionnaire is employed to collect respondents’ opinions on the degree of relevance of each category of information to transform the current level of practice into a more advanced and simplified approach. The degree of information relevance used a weighted average of the three cases and each category of information is analysed and shown in Table 5, which describes the information requirements in six categories based on the structured interview analysis. A summary of the degree of information relevance using the weighted average of the three cases and each information category is analysed using equation (1) and equation (2) and presented in Fig. 6. The details of the responses on the degree of importance of information requirements in six categories from the structured interview are shown in Table 5.

**Table 5:** Degree of information relevance for built asset information modelling.

No	Information Requirements	Weighted Mean			Average
1	Basic Project Information	Case1	Case 2	Case 3	
1.1	Asset name	9.25	9.67	10.00	**9.64**
1.2	Location	8.75	9.67	10.00	**9.47**
1.3	Employer	9.25	9.67	10.00	**9.64**
1.4	Contractor	8.75	9.33	10.00	**9.36**
1.5	Consultant	9.25	9.33	10.00	**9.53**
1.6	Date of start	8.75	9.33	10.00	**9.36**
1.7	Date of asset handover	9.25	9.67	10.00	**9.64**
1.8	Cost at completion	8.75	9.67	10.00	**9.47**
1.9	Number of floors	9.25	9.67	10.00	**9.64**
1.1	Type of contract	8.25	8.67	9.50	**8.81**
1.11	Project delivery method	8.25	9.00	9.50	**8.92**
1.12	Function of the built asset	8.75	9.33	9.50	**9.19**
1.13	Technical Characteristics (Classic, Modern and contemporary)	8.25	9.33	9.50	**9.03**
**2**	**Technical Information**				
2.1	Software platforms	8.75	8.67	9.00	**8.81**
2.2	Data Exchange Formats	8.50	8.33	9.00	**8.61**
2.3	Visualization format	9.00	8.33	9.00	**8.78**
2.4	Classification of system of BIM data	8.75	8.67	9.00	**8.81**
2.5	Coordinate points	9.00	8.67	9.00	**8.89**
2.6	Level of development	8.75	9.00	9.50	**9.08**
2.7	Mechanical, Electrical and Plumbing Systems	8.50	9.33	10.00	**9.28**
2.8	Training details	8.50	9.00	10.00	**9.17**
2.9	Engineering data and design parameters	8.75	9.33	10.00	**9.36**
2.1	Asset dependencies and interdependencies	9.00	9.33	10.00	**9.44**
2.11	Date of commission and data submitted	8.25	9.00	10.00	**9.08**
2.12	Operational data, performance characteristics and design limits	8.75	9.33	10.00	**9.36**
**3**	**Managerial Information**	**Case1**	**Case 2**	**Case 3**	
3.1	Asset type	9.00	9.33	10.00	**9.44**
3.2	Photograph	9.00	8.67	10.00	**9.22**
3.3	Identification numbers	8.50	8.33	10.00	**8.94**
3.4	Location	9.25	8.67	10.00	**9.31**
3.5	Floor area	9.25	9.33	10.00	**9.53**
3.6	Space management information	9.00	9.33	10.00	**9.44**
3.7	Warranties and guarantee periods	9.25	9.33	9.50	**9.36**
3.8	Access planning and work schedules	9.00	9.00	9.00	**9.00**
3.9	Maintenance and inspection schedules and records	8.75	8.67	9.00	**8.81**
3.10	Operation and maintenance manual	8.75	8.33	9.00	**8.69**
3.11	Outstanding tasks	9.00	9.33	9.00	**9.11**
3.12	Record of planned and unplanned maintenance	9.25	9.00	9.00	**9.08**
3.13	Standards, processes, and procedures	9.25	9.33	9.50	**9.36**
3.14	Hazardous contents or waste	8.75	8.67	9.50	**8.97**
3.15	End of the life cycle of the asset	9.25	9.00	9.50	**9.25**
3.16	Emergency plans	9.25	9.33	9.50	**9.36**
3.17	The presence of any hazardous contents and waste	9.25	9.00	9.50	**9.25**
3.18	Details of emergency plans including responsibilities and contact	9.25	9.00	9.50	**9.25**
3.19	Linked enterprise data information related to specific building asset.	9.25	9.33	10.00	**9.53**
3.20	User's Manual	9.00	8.67	10.00	**9.22**
**4**	**Commercial Information**				
4.1	Asset description	8.50	9.00	9.50	**9.00**
4.2	Asset function	8.75	8.33	9.50	**8.86**
4.3	Supplier's detail	8.75	9.00	9.50	**9.08**
4.4	Lead time	8.75	9.67	9.50	**9.31**
4.5	Condition of facility and equipment	8.75	8.67	9.50	**8.97**
4.6	Key performance indicators	8.75	9.33	9.50	**9.19**
4.7	Performance targets or standards	9.00	9.00	9.00	**9.00**
4.8	Non-conformance criteria and actions to be taken	9.00	9.33	9.50	**9.28**
4.9	The criticality of assets and spaces to the organization	8.75	9.00	9.50	**9.08**
**5**	**Financial Information**				
5.1	Initial purchase/leasing Cost	9.50	8.67	9.50	**9.22**
5.2	Operational cost	9.50	9.00	9.50	**9.33**
5.3	Cost of planned maintenance	9.50	9.33	9.50	**9.44**
5.4	Historical maintenance cost	9.25	9.00	9.50	**9.25**
5.5	Asset replacement value	9.00	9.33	9.50	**9.28**
**6**	**Legal Information**				
6.1	Ownership details	9.50	9.67	10.00	**9.72**
6.2	Maintenance demarcation	9.50	9.33	10.00	**9.61**
6.3	Contractual information	9.25	9.00	9.00	**9.08**
6.4	Property boundaries	9.75	9.00	9.50	**9.42**
6.5	Work instructions	9.50	9.00	9.00	**9.17**
6.6	Legal obligations (H&S, etc)	9.00	9.33	9.50	**9.28**
6.7	Risk assessments and control measures	9.00	9.00	9.00	**9.00**

**Average values from 8.10 to 10 are identified as the most relevant information.

From the results presented in Table 5 and it is evident that the absence of adequate information within the built asset management division of public built assets in universities of the selected case studies is significantly affecting the performance and decision-making process. Consequently, the interviewees underscored the significance of nearly all the specified information (outlined in the question guide of the interview) in their decision-making procedures. Nevertheless, it appears excessively ambitious to provide all the requested data to the BAM department because of resource limitations. This requires a criteria-based selection of information categories and subcategories for efficient generation of information on existing buildings.

### 4.4 Conceptual framework for BIM-enabled BAM

Collection of electronic and manual information for existing buildings to support O&M activities often result in the wastage of time and effort of asset managers. This results in a redundancy of activity in searching, sorting, validating, and recreating built asset data/information within the asset management department. The application of BIM-enabled asset information management for O&M functions can exhibit the ability to provide greater precision and accuracy throughout an asset lifecycle. This eliminates the costs associated with data entry while producing high quality data, overcoming the challenges of data retrieval, organisation, storage, and interoperability. The present study has developed a BIM enabled asset information management conceptual framework designed to address challenges posed by Ethiopia's prevailing practice and managing data and information related to the built asset.

**Fig. 7:**
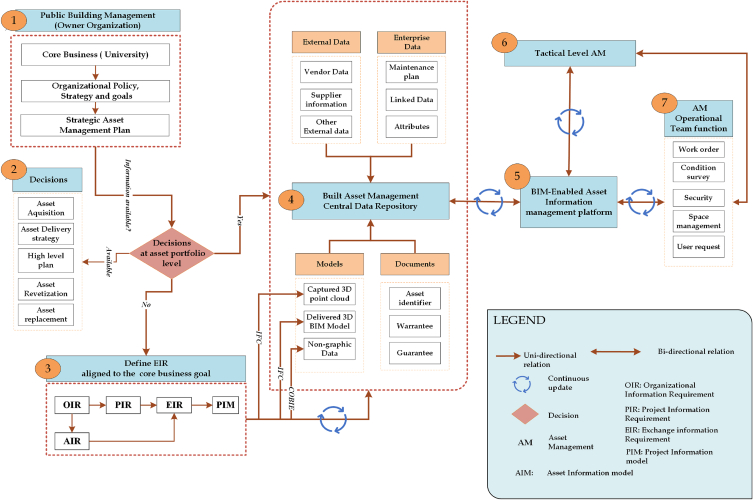
Conceptual framework for BIM-Enabled built asset information management.

The present study strategizes for developing a unified platform, as the assets of a public university must serve as the single source of accurate data and/or information. Such a platform could effectively enhance the management of asset data and facilitate project data and/or information delivery during asset handover. Then, it integrates the functionalities and processes for supporting and checking asset information requirements (AIRs) throughout the life cycle of an asset. It also considers the involvement of diverse decision-makers throughout the asset life cycle in the provision of necessary information for BIM-enabled asset management. The framework delineates the establishment of structured information requirements and the validation of various data sources across the asset life cycle. This study focuses on advancing existing built asset information management practices shown in Fig. 7 to a higher level of maturity using a systematic approach while leveraging BIM. The framework is tailored to accommodate various types of asset data (such as files, databases, documents, drawings, and models). It allows users to progress from basic database management to sophisticated asset information management platforms.

The framework is also designed to process managing, organizing, storing, and protecting project information in a way that is accessible at any point in time in an organized manner to minimize time wasted in searching for asset data/information. As there are diverse sources for entering information into the central repository (component 4), the issue of data security, authorized access, and data modification could be considered a challenge. However, technological advancements in the IT sector, such as block chain, have provided data security solutions in modelling asset information, as explained by Raslan et al. [16]. The developed conceptual framework is shown in Fig. 7.

## 5 Discussion

### 5.1 Discussion on the cross-cases

The built asset management departments within the Ethiopian public universities lack an asset registry database for the purpose of storing and managing built asset data and its corresponding documents. This situation has led to a scarcity of as built documents for existing buildings. However, it is worth noting that a project office within the owner organization of the present study possesses (responsible for the construction process) a type of as-built documents for recently completed projects. This deficiency has adversely affected the cost, schedule, and duration of operation and maintenance (O&M) performance. In addition, it has disrupted the monitoring and control processes of the O&M team.

Currently, the BAM department relies on the traditional (manual) method of collecting, visualising, and managing data from existing buildings. The current approach for transferring as-built data is based on printed paper documents (hardcopy), scanned documents, AutoCAD documents, Word documents and Excel documents as the predominant formats for data transfer from the property developer to the owner/BAM department during asset handover. In addition, BAM departments lack a standardised data model and building/data classification system for managing building information, which in turn affects the performance of the operations team. Consequently, there is a need to upgrade current practises to a more advanced level in order to move towards data-driven decision making [42,93].

As discussed previously, the operation and maintenance phase (O&M) has been identified as the most expensive phase in a building's life cycle [9]. This cost can potentially range from 60 to 85 % of the total lifecycle cost [27,33,40,94,95]. Within the current study's context, the cost associated with rework and time taken to find information from existing buildings using conventional approaches have not been adequately studied. As a developing economy with constraints of financial resources, a strong suggestion is put forth to apply the utmost attention for achieving the most valuable outcomes from built assets. It can be said that there is limited knowledge of technological advances such as sensors, scanners, and detectors for fault detection techniques to conduct maintenance activities efficiently and effectively.

Built-asset data within an asset management system can be communicated from upstream for downstream use [7]. However, for the effective functioning of operational teams, asset graphic data and information required to specific purposes and at a project level shall be updated to reflect changes, which remains unaddressed. Interoperability has also been a key challenge in data transfer and is a costly endeavor [96]. In prior practice, Gallaher et al. [97] Mirarchi [97] et al. argue that the challenges of interoperability can cost a capital built asset industry around $15.8 billion annually.

The cases included in the present study pertain to the predominant practices of BAM, which employs a traditional methodology, characterized by the absence of paper-based documentation. As a result, the BAM practice requires the implementation of robust technologies to improve the deep-rooted challenges. In line with this, built asset management department have shown interest to adopt emerging processes and technologies like Building Information Modelling (BIM) in congruence with [98]. The emergence of these processes and technologies are used to manage information throughout an asset lifecycle and improve BAM efficiency [45]. Similarly, Hossain & Yeoh [5] stated that BIM-based BAM platforms play a significant role in improving O&M performances.

Regarding the information requirement, there is increasing evidence for business value of BIM in asset management. In spite of its business benefits, the information requirement for operation activities has been a subject of extensive study [9,20,50]. Following an extensive research, operational information requirement is identified and a standard document is created: PAS1992–3:2014 for use in the UK industry, which later was changed to EN ISO 19650–3:2018 [63] as a global standard for information requirement for BIM-based asset information modelling.

However, it is still difficult to define what specific information is required by built asset management department to effectively perform their tasks appears theoretical, (Ashworth et al. [20] and difficult to implement and the suitability of the approach is not mutually agreed among built asset management domain [7]. Hence, information requirement needs to be re-defined considering the basis of the business value, process maturity, technology maturity and economic situation of the context. In line with this, the present study found information required by public university-built asset management department to effectively manage operational functions in specific situations.

As explained in Table 5, BAM department requires possessing information pertaining to six distinct categories to effectively execute operational tasks. However, this finding can be interpreted in two ways. The first viewpoint being a BAM department's performance is hindered by a lack of information in all categories, while a second perspective is that the interviewees are unaware of the costs and time-consuming nature of generating all categories of information from existing buildings. Accordingly, the authors argue to establish a criterion-based information requirement that facilitates the acquisition of information critical to the decision-making process of the department.

### 5.2 Discussion on the conceptual framework

The conceptual framework developed in this study aims to represent the key process, the diverse information sources, the data hub, the business organization, and other stakeholders involved in the process and their interaction. Mapping all processes, actors, decisions, and interactions in one framework helps users easily grasp the key components of the framework and use it to automate the process using the approach shown in Fig. 7.

The BIM-Enabled asset information modelling conceptual framework developed in the present study is depicted in Fig. 7, comprising seven main components. Each component of the conceptual framework is described below.

• ***Component 1:*** The primary step is to recognize and acknowledge the mission, vision, goal/objectives, policies, and strategy of core organization (in this case a public university). Upon the recognition, a strategic built asset management plan must be devised that aligns with the core business objectives. This strategic plan is set out in such a manner that organizational data and information requirements are defined; decisions processes are defined; and process of exchange information requirement (EIR) are explained. In this phase, the assumption is that built asset management department is established and integral part of the organizational structure, which illustrates the level of awareness, knowledge, maturity and need of an organization.
• ***Component 2:*** This part represents the possible decisions made at the asset portfolio level, which depends on the availability of asset information. At a strategic level, possible decisions are identified, and the type of information needs to be defined to support a data-driven decision-making process. If data is required to make an informed decision and is not found, the decision could be the generation of information either for asset acquisition, asset delivery, a high-level plan, asset revitalization, or asset replacement as a key decision in the operational phase.
• ***Component 3:*** Represents the definition of information requirements following the owner's strategic objectives and as defined in ISO19650-3. The requirements should be integrated with potential decisions and the requirements of the central repository.
• ***Component 4:*** It is the central data repository, which serves as a hub for a wide array of heterogeneous data originating from various sources and formats. This repository provides the necessary inputs for developing the primary asset data model. In this component, all pertinent data and information identified in the preceding stages are stored to enable interoperability, facilitate seamless sharing among all relevant stakeholders and ensure data security policy.
• ***Component 5:*** It consists of a primary Asset information management platform that extracts verified data from the central repository to execute automated day-to-day operational phase functions intended to improve the primary organizational performance objectives and bring excellence.
• ***Component 6:*** This element of the framework pertains to the decision-making module of the operational phase functions team leader and its integration with the asset management platform by both the strategic asset management plan and the organizational core objectives. In this module, communication follows among tactical-level operational team members and their leaders through an automated process to execute tasks with continuous information updates.
• ***Component 7:*** This aspect refers to the concerns of recognizing functions within the operational team and their automated interaction with higher-level management to improve the overall performance of core business activities. Additionally, the platform provides the ability to update information related to executed tasks, thus simplifying reporting, filing, and monitoring activities.

In developing the framework, the findings from local practice integrated with various standards and specifications have been used to streamline the structured development of owner information requirements (EIR) and their validation against various data sources. The EN-ISO 19650 series specifications are used as a source of information in developing the conceptual framework, while the work of [99] is used as reference material in re-shaping the framework. The standard series outlines essential guidelines for managing information throughout the asset's operational phase, incorporating various data sources and relevant technologies. This framework aims to transform operational teams from conventional/traditional workflows to digital solutions in public building asset management. Ultimately, the study's findings have the potential to elevate asset management practices in public buildings in Ethiopia.

## 6 Conclusion

This study explored the current built asset information management practices in public buildings and highlighted the key requirements for effective asset management enabled by BIM. The study revealed a worrying reality: data on existing buildings transferred from the development phase is often non-existent, incomplete, inaccurately documented or of limited use for operational purposes. These findings emphasize the urgent need for strategic action to raise the standards of asset information management practice in Ethiopia's public buildings. Without reliable and accurate information, operational teams are hindered in their ability to effectively perform built asset management tasks, compromising the core competencies of towner organizations.

Therefore, it is essential to describe the information requirements for BIM-enabled built asset management to empower the operational teams in the owner organizations. A conceptual framework has been developed to guide the management of building information using BIM and provide a way to not only improve existing practices but also enhance overall performance. This framework has been developed to streamline the storage, retrieval and sharing of information across different data sources. It includes as-built BIM models and enterprise-linked data, documents, all based on an open data format.

The implications of this study go beyond the boundaries of the specific challenges identified in its context. Rather, the findings are promising when it comes to overcoming the persistent difficulties associated with the availability, accuracy, and reliability of information in broader areas. By adopting the findings of the study, stakeholders are able to overcome these challenges and lead in an era of improved operational efficiency and informed decision making in the area of built asset management.

## Recommendation

Further studies are recommended to figure out the information requirement during the operation & maintenance phase based on certain criteria and for specific decisions for efficient creation of information in built asset information modelling. The inclusion of multiple cases may enhance the results and improve replication of the findings for similar situation.

## Contribution to the literature

The conceptual framework developed identified key processes, information sources and flows and their integration into BIM-based asset information management. It also raises awareness regarding the importance of utilising technological developments to solve challenges in asset information modelling and paves the way to data-driven decision-making process.

## Limitation of the study

The results of this study are based on expert opinion and a case study in a specific scenario, which may affect replication for similar situations due to subjectivity. The study lacks to demonstrate a typical digital built asset information model based on the developed conceptual model.

## Data availability

The primary and secondary data used to support this study are available from the corresponding author up on request.

## Funding statement

This study was not financially supported by any organization or institution.

## CRediT authorship contribution statement

**Muluken Tilahun Desbalo:** Writing – review & editing, Writing – original draft, Methodology, Formal analysis, Data curation, Conceptualization. **Asregedew Kassa Woldesenbet:** Writing – review & editing, Validation, Supervision. **Tamiru Mengist Habtu:** Data curation, Formal analysis, Writing – review & editing. **Hans-Joachim Bargstädt:** Project administration, Validation, Visualization, Writing – review & editing. **Mitiku Damtie Yehualaw:** Writing – review & editing, Visualization, Validation.

## Declaration of competing interest

The authors declare that they have no known competing financial interests or personal relationships that could have appeared to influence the work reported in this paper.
